# Digitale Gesundheitsanwendungen, Telemedizin und Large Language Models in der Dermatologie: Ergebnisse einer deutschlandweiten Befragung

**DOI:** 10.1111/ddg.70019

**Published:** 2026-10-05

**Authors:** Lukas Henk, Leah Schirren, Felix Mühlensiepen, Claudia Traidl‐Hoffmann, Marina Otten, Victor Olsavszky, Alexander Zink, Michael Hertl, Sebastian Kuhn, Johannes Knitza

**Affiliations:** ^1^ Institut für Digitale Medizin Universitätsklinikum Gießen und Marburg Philipps‐Universität Marburg Marburg Deutschland; ^2^ Klinik für Dermatologie und Allergologie Philipps‐Universität Marburg Marburg Deutschland; ^3^ Zentrum für Versorgungsforschung Brandenburg Medizinische Hochschule Brandenburg Theodor Fontane Neuruppin Deutschland; ^4^ Institut für Umweltmedizin und integrative Gesundheit Medizinische Fakultät Universitätsklinikum Augsburg Augsburg Deutschland; ^5^ CK‐Care, Christine Kühne Center for Allergy Research and Education Davos Schweiz; ^6^ Institut für Versorgungsforschung in der Dermatologie und bei Pflegeberufen Universitätsklinikum Hamburg‐Eppendorf Hamburg Deutschland; ^7^ Klinik für Dermatologie Venerologie und Allergologie Universitätsklinikum Mannheim Medizinische Fakultät Mannheim der Universität Heidelberg Mannheim Deutschland; ^8^ Klinik für Dermatologie und Allergologie Technische Universität München München Deutschland

**Keywords:** DiGA, Telemedizin und LLM in der Dermatologie, ChatGPT, eHealth, mobile applications, large language models, Telemedicine

## Abstract

**Hintergrund:**

Digitale Technologien bieten Chancen für die dermatologische Versorgung, doch fehlen systematische Daten zur Nutzung und Akzeptanz, insbesondere zu digitalen Gesundheitsanwendungen (DiGA) und Large Language Models (LLMs).

**Patienten und Methodik:**

Von November 2024 bis Januar 2025 wurde eine anonyme Online‐Befragung unter Dermatolog*innen im Rahmen von Fortbildungen durchgeführt. Erfasst wurden Nutzung und Einstellungen zu Telemedizin, DiGA und LLMs sowie Kompetenzeinschätzungen und Fortbildungsbedarf.

**Ergebnisse:**

100 Dermatologen nahmen teil (64% weiblich, Durchschnittsalter 40,6 Jahre). 58% gaben an, derzeit keine telemedizinischen Leistungen anzubieten; lediglich 5 % nutzten regelmäßig Videosprechstunden. 21% hatten bereits DiGA verordnet, wobei die Mehrheit (78%) die Verantwortung dafür bei Allgemeinärzt*innen sah. 96% der Dermatolog*innen waren der Ansicht, dass DiGA die medizinische Versorgung verbessern können. Als Hauptbarrieren wurden mangelndes Wissen (76%), fehlende Zeit (74%) sowie unzureichende Vergütung und ein komplexer Verschreibungsprozess (je 43%) genannt. 45% der Befragten hatten LLMs im beruflichen Kontext genutzt. 76% bewerteten die Auswirkungen der Digitalisierung auf die Patientenversorgung als positiv und 80% äußerten den Wunsch nach Fortbildungsangeboten zum Einsatz digitaler Technologien im ärztlichen Alltag.

**Schlussfolgerung:**

Die Digitalisierung und insbesondere DiGA werden von Dermatologen überwiegend positiv wahrgenommen, digitale Angebote werden jedoch bislang kaum genutzt. Ein gezielter Wissensausbau und konkrete Fortbildungsangebote könnten die Implementierung digitaler Technologien im dermatologischen Alltag wesentlich fördern.

## EINLEITUNG

Digitale Technologien prägen zunehmend die medizinische Versorgung und bieten insbesondere in der Dermatologie vielfältige Möglichkeiten zur Optimierung von Diagnostik und Therapie.^1^, ^2^ Mit der Veröffentlichung der ersten S2k‐Leitlinie zur Teledermatologie im Jahr 2020 war die Dermatologie das erste medizinische Fachgebiet in Deutschland, das sich im Rahmen einer evidenzbasierten Leitlinie systematisch mit telemedizinischen Versorgungsmodellen auseinandersetzte.^3^ Ergänzend formulierten die Deutsche Dermatologische Gesellschaft (DDG) und der Berufsverband der Deutschen Dermatologen (BVDD) im Jahr 2023 in einem gemeinsamen Positionspapier Perspektiven zur Weiterentwicklung digitaler Versorgungsstrukturen in der Dermatologie, wobei insbesondere die Integration von Gesundheits‐Apps und Künstlicher Intelligenz sowie die Notwendigkeit spezifischer ärztlicher Qualifizierungsangebote hervorgehoben wurden.^4^

Eine Querschnittsbefragung aus den Jahren 2019 und 2021 dokumentierte eine Zunahme teledermatologischer Videosprechstunden während der COVID‐19‐Pandemie.^5^ Aktuell sind 69 Digitale Gesundheitsanwendungen (DiGA) gelistet (Stand: Juli 2025), von denen bislang keine explizit für dermatologische Indikationen zugelassen ist. Einige Anwendungen adressieren jedoch relevante Komorbiditäten wie Depression, Raucherentwöhnung, arterielle Hypertonie oder Adipositas.^6^, ^7^ DiGA werden von allen gesetzlichen Krankenkassen und von vielen privaten Krankenversicherungen erstattet. Die umfassende Prüfung von Sicherheit und Nutzen durch das Bundesinstitut für Arzneimittel und Medizinprodukte (BfArM) bietet sowohl Patienten als auch Ärzten einen entscheidenden Vorteil gegenüber den sonst häufig wechselnden und intransparenten App‐Angeboten. Parallel dazu gewinnen Large Language Models (LLMs) wie ChatGPT zunehmend an Bedeutung. Potenzielle Einsatzbereiche reichen von der Unterstützung bei der Anamneseerhebung, die Generierung von Arztbriefen, die Formulierung von Patienteninformationen bis hin zur strukturierten Auswertung von Leitlinien.^2^ In welchem Umfang die Videosprechstunde, DiGA und LLMs von Dermatologen genutzt werden, ist bislang unklar.

Ziel dieser Studie war es daher, systematisch die Nutzung, Akzeptanz und wahrgenommenen Barrieren digitaler Technologien in der Dermatologie zu erfassen. Der Schwerpunkt lag insbesondere auf insbesondere DiGA und LLMs. Darüber hinaus wurde der Fortbildungsbedarf dermatologisch tätiger Ärzt*innen im Umgang mit digitalen Technologien erhoben.

## METHODEN

Die Datenerhebung erfolgte digital mittels eines anonymen Online‐Fragebogens (Google Forms). Die Ergebnisse wurden unter Berücksichtigung der CHERRIES‐Empfehlungen (Checklist for Reporting Results of Internet E‐Surveys) strukturiert berichtet.^8^ Dermatologen wurden durch Studienpersonal im Rahmen von dermatologischen Fortbildungsveranstaltungen (Derma Update‐Kongress, Berlin, sowie lokale Fortbildungen am Universitätsklinikum Marburg) zwischen November 2024 und Januar 2025 rekrutiert. Die Teilnehmenden konnten den Fragebogen entweder direkt auf einem bereitgestellten Tablet ausfüllen oder über einen QR‐Code auf ihrem eigenen Gerät bearbeiten. Die Teilnahme erfolgte freiwillig und ohne finanzielle oder materielle Entschädigung. Es wurden nur vollständig ausgefüllte Fragebögen in der Auswertung berücksichtigt. Die Ethikkommission der Philipps‐Universität Marburg bestätigte, dass für diese anonyme Erhebung keine Genehmigung erforderlich ist (24‐292 ANZ).

Der Fragebogen umfasste insgesamt 16 Fragen (Online Supplement S1). Neben soziodemografischen Merkmalen wurde die Nutzung von telemedizinischen Anwendungen und LLMs abgefragt. Erfasst wurden zudem die Selbsteinschätzung digitaler Kompetenzen, der individuelle Fortbildungsbedarf sowie die Wahrnehmung der Auswirkungen der Digitalisierung auf die Patientenversorgung. Ein besonderer Schwerpunkt lag auf digitalen Gesundheitsanwendungen (DiGA), zu denen sechs Fragen gestellt wurden, die Nutzung, Akzeptanz und wahrgenommene Barrieren erfassten.

Zur Datenanalyse wurden Microsoft Excel für Mac (Microsoft Corporation, Redmond, WA, USA) und IBM SPSS Statistics, Version 30.0.0.0 (IBM Corporation, Armonk, NY, USA) verwendet. Zusammenhänge zwischen ordinalskalierten Variablen wurden mittels Rangkorrelation nach Spearman (Spearman‐Rho) analysiert. Die Ergebnisse werden im Text als ρ = [Korrelationskoeffizient], p = [p‐Wert] angegeben.

## ERGEBNISSE

### Demografische Daten

Insgesamt beantworteten 100 Teilnehmende den Fragebogen, von diesen identifizierten sich 64% als weiblich und 36% als männlich. Das durchschnittliche Alter lag bei 40,6 Jahren. Der Großteil der Teilnehmenden arbeitete an Universitätskliniken (44%), gefolgt von Einzelpraxen (28%) und Gemeinschaftspraxen (18%) (Tabelle 1).

**TABELLE 1:** Demografische Daten.

Merkmale	Befragte
**Geschlecht, n (%)**	
Weiblich	64 (64)
Männlich	36 (36)
Divers	0
**Alter (Jahre), n (%)**	
20‐30	23 (23)
31‐40	34 (34)
41‐50	22 (22)
51‐60	13 (13)
>61	8 (8)
**Arbeitsposition, n (%)**	
Einzelpraxis	28 (28)
Gemeinschaftspraxis	18 (18)
MVZ	4 (4)
Klinikum der Grund‐ und Regelversorgung	1 (1)
Klinikum der Maximalversorgung	5 (5)
Universitätsklinikum	44 (44)

### Nutzung von Telemedizin

Mit Blick auf den Anteil telemedizinisch versorgter Patientinnen und Patienten gaben 58% der Befragten an, aktuell keine telemedizinischen Leistungen anzubieten. Weitere 27% nutzen Teledermatologie für weniger als 10% ihrer Patientinnen und Patienten. Lediglich 12% gaben an, einen höheren Anteil (10% bis 50%) telemedizinisch zu betreuen. Die am häufigsten genutzten Formen telemedizinischer Anwendungen betrafen die asynchrone Kommunikation unter Kollegen (70% „oft“ oder „eher oft“), die synchrone Kommunikation unter Kollegen (54% „oft“ oder „eher oft“) sowie die asynchrone Kommunikation mit Patienten (54% „oft“ oder „eher oft“). Videosprechstunden wurden von 5% der Befragten „oft“ oder „eher oft“ eingesetzt (Abbildung 1). Unter asynchroner Kommunikation waren beispielsweise Konsilanfragen oder E‐Mails zu verstehen, während mit synchroner Kommunikation vor allem Telefonate oder Videokonferenzen gemeint waren.

**ABBILDUNG 1:**
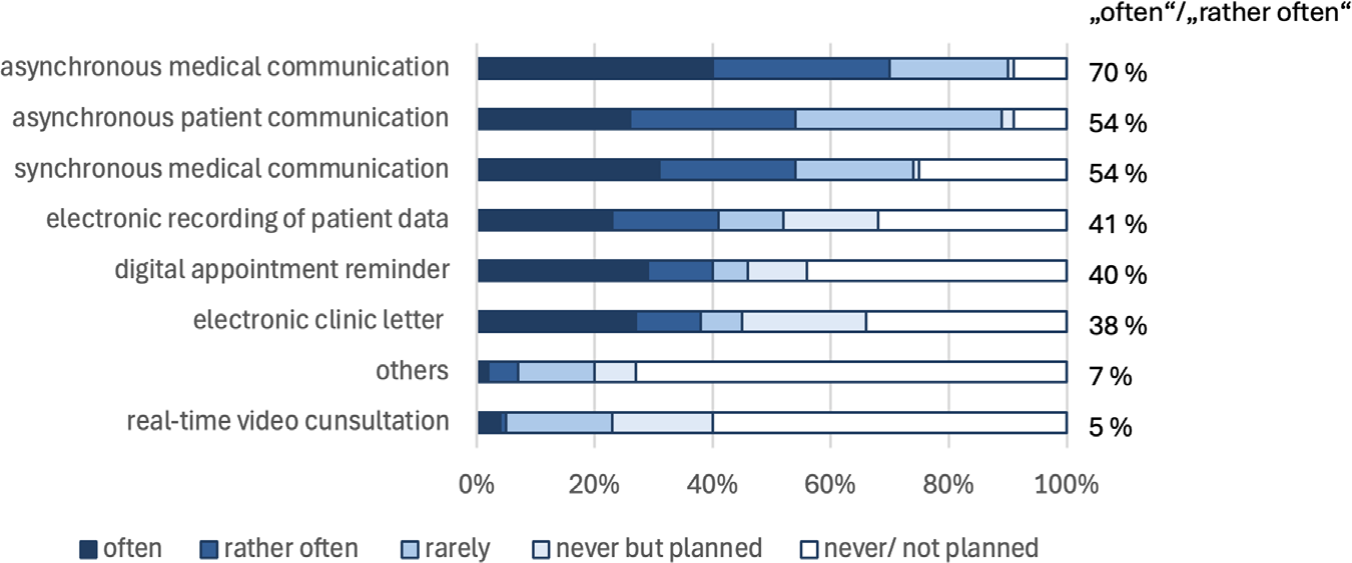
Nutzung von digitalen Tools in der klinischen Routine.

### Akzeptanz, Nutzung und Barrieren im Umgang mit DiGA

96% der befragten Dermatologen sahen in DiGA allgemein ein großes Potenzial, die Versorgung zu verbessern. Obwohl derzeit noch keine DiGA speziell für dermatologische Indikationen zugelassen sind, sähen 97% ein Potenzial in spezifisch dermatologischen DiGA. 21% der befragten Dermatologen gaben an, bereits mindestens einmal eine DiGA verordnet zu haben. Von diesen verordnen 14% DiGA etwa einmal pro Monat, 2% etwa wöchentlich und 5% nahezu täglich. Die Verantwortung für die Verordnung wird überwiegend bei Allgemeinärzten gesehen (78%), gefolgt von anderen Fachärzten (63%) (Abbildung 2). Das eigene Wissen zu digitalen Gesundheitsanwendungen wurde von den meisten Teilnehmenden als eher gering eingeschätzt (4/10; IQR: 3). Dieses begrenzte Wissen wurde zugleich am häufigsten als Barriere bei der Verordnung genannt (76%), gefolgt von fehlender Zeit für Aufklärung und Einweisung (74%), unzureichender Vergütung sowie einem als kompliziert empfundenen Verschreibungsprozess (jeweils 43%) (Abbildung 3).

**ABBILDUNG 2:**
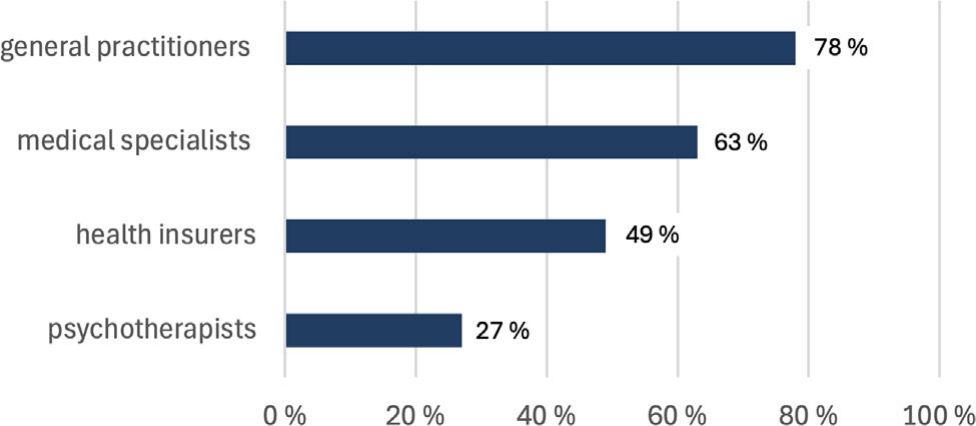
Zuständigkeit für Verschreibung und Information über DiGA aus Sicht der Befragten (Mehrfachantworten möglich).

**ABBILDUNG 3:**
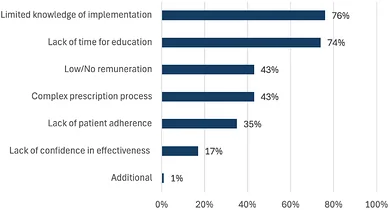
Wahrgenommene Barrieren bei der Verschreibung von DiGA (Mehrfachauswahl möglich).

### Nutzung von Large Language Modellen

45% der Dermatologen nutzten LLMs im beruflichen Kontext, davon 25% monatlich, 14% wöchentlich und 6% täglich (Abbildung 4). Die Korrelationsanalyse ergab eine moderate, signifikante negative Korrelation zwischen dem Alter der Teilnehmenden und der Nutzung im Arbeitskontext (ρ = ‐0,417, p < 0,001).

**ABBILDUNG 4:**
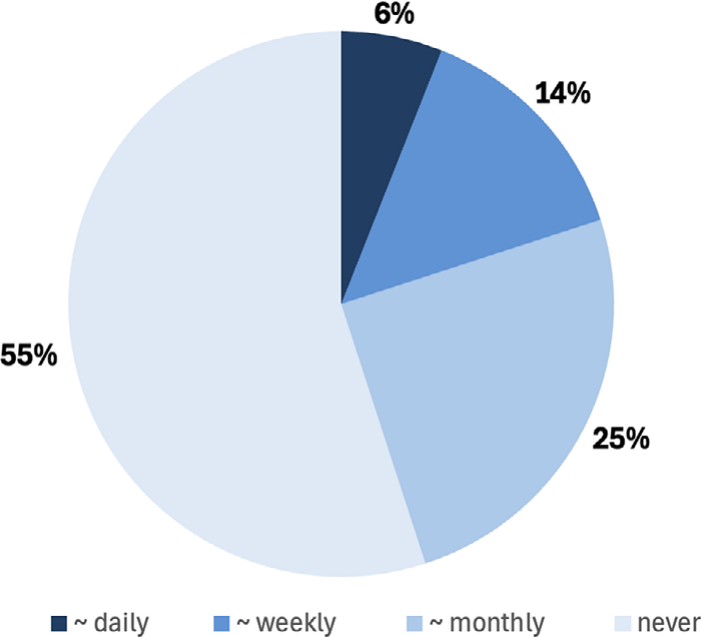
Nutzung von Large Language Models im Rahmen der ärztlichen Tätigkeit.

### Einstellungen, Kompetenzeinschätzungen und Fortbildungsbedarf zu digitaler Medizin

76% der Befragten bewerteten den Einfluss der Digitalisierung auf die Patientenversorgung als (sehr) positiv, nur 1% als (sehr) negativ, der Rest gab eine neutrale Bewertung ab. Von den Befragten schätzten 42% ihre Kenntnisse in der digitalen Medizin als gut ein und 23% ihre Kenntnisse als nicht ausreichend. In Bezug auf die Sicherheit im Umgang mit medizinischen digitalen‐ und Onlineanwendungen schätzten 56% diese als gut ein, während 12% sich im Umgang unsicher fühlen. In einer Pearson‐Korrelationsanalyse zeigte sich kein signifikanter Zusammenhang zwischen dem Alter der Teilnehmenden und ihrer selbsteingeschätzten Kenntnis im Bereich der digitalen Medizin (ρ = 0,046, p = 0,65) oder ihrer empfundenen Sicherheit im Umgang mit medizinischen digitalen Anwendungen (ρ = 0,011, p = 0,91). 80% der Befragten würden Schulungen beziehungsweise Fortbildungen zum Einsatz digitaler Tools in der klinischen Praxis begrüßen.

## DISKUSSION

Die Ergebnisse dieser Studie zeigen, dass digitale Technologien, insbesondere DiGA, von Dermatologen überwiegend positiv bewertet werden. Dennoch findet eine konkrete Nutzung telemedizinischer Verfahren nur in begrenztem Umfang statt. Die geringe Verordnungshäufigkeit von DiGA und die geringe Nutzung von Videosprechstunden unterstreichen die Diskrepanz zwischen theoretischer Akzeptanz und praktischer Anwendung. Im Vergleich zu früheren Erhebungen, die während der COVID‐19‐Pandemie eine erhöhte Nutzung von Videosprechstunden dokumentierten, zeigt sich in unseren Daten erneut ein Rückgang dieser Anwendung.^5^ Dieser Trend ließ sich ebenfalls in einer Umfrage aus dem Jahr 2023 nachweisen, die unter Ärzten durchgeführt wurde, die Patienten mit chronischer Urtikaria betreuten.^9^

Ein zentrales Ergebnis der Umfrage war die geringe Verordnungsrate von DiGA, obwohl 96% der Teilnehmenden deren potenziellen Nutzen für die medizinische Versorgung anerkannten. 21% gaben an, bereits eine DiGA verordnet zu haben. Dieser Anteil liegt laut Vergleichsstudien deutlich unter den Verordnungsraten in anderen Fachgebieten wie der Allgemeinmedizin oder der Inneren Medizin.^10^, ^11^ Die geringe Verordnungshäufigkeit könnte vor allem auf das Fehlen zugelassener DiGA für spezifisch dermatologische Indikationen zurückzuführen sein. Derzeit beschränkt sich der Einsatz in der Dermatologie auf DiGA zur Behandlung begleitender Komorbiditäten. Gerade bei chronischen Erkrankungen wie Allergien, Psoriasis oder Akne inversa, bei denen Lebensstilfaktoren und aktives Selbstmanagement eine zentrale Rolle spielen, könnten DiGA einen wichtigen Beitrag zur Unterstützung der Versorgung leisten.^12^, ^13^, ^14^, ^15^

Ein zentrales Hemmnis stellt das als unzureichend eingeschätzte Wissen der Befragten zu digitalen Gesundheitsanwendungen dar, das auch als häufigste Barriere für die Verordnung angegeben wurde. Diese Befunde decken sich mit Studien aus anderen Fachgebieten, in denen mangelnde Information, begrenzte Zeitressourcen sowie unklare Vergütungsstrukturen als wesentliche Hürden identifiziert wurden.^11^, ^16^, ^17^

Auch wenn die Integration von LLMs in die medizinische Versorgung noch am Anfang steht, zeigt die vorliegende Befragung, dass diese vergleichsweise häufig bereits von vielen Dermatologen im beruflichen Alltag genutzt werden. Zukünftige Studien sollten untersuchen, in welchen konkreten Anwendungsbereichen LLMs bereits zum Einsatz kommen und welche Potenziale sowie Herausforderungen sich dabei in der dermatologischen Praxis ergeben. Auffällig war eine signifikante negative Korrelation zwischen dem Alter der Teilnehmenden und der Nutzungshäufigkeit. Gezielte Fortbildungsangebote könnten dazu beitragen, dass diese Technologien auch von älteren Kollegen häufiger und adäquat genutzt werden.

Der von 80% der Befragten geäußerte Wunsch nach Fortbildungsangeboten in digitaler Medizin unterstreicht den bestehenden Qualifizierungsbedarf. Inhalte zum Thema digitale Medizin sind in der aktuellen Musterweiterbildungsordnung (MWBO) bislang nur unzureichend berücksichtigt. Fachgesellschaften wie die Deutsche Dermatologische Gesellschaft (DDG) und der Berufsverband der Deutschen Dermatologen (BVDD) haben in einem gemeinsamen Positionspapier bereits konkrete Forderungen formuliert. Darin betonen sie, dass die Qualifizierung von Ärzten im Umgang mit digitalen Verfahren „deutlich stärker als bisher in die Aus‐, Weiter‐ und Fortbildung implementiert werden“ müsse.^4^ Diese Forderung spiegelt sich unter anderem im Zertifikat „Teledermatologie“ der Deutschen Dermatologischen Akademie (DDA) wider, das Ärzten gezielt für telemedizinische Konsile schult. Ergänzende Fortbildungsangebote zu DiGA und LLMs könnten hier eine sinnvolle Erweiterung darstellen. Auch skalierbare Onlineformate wie die Webinare des Digitale Dermatologie e. V. bieten bereits niedrigschwellige Zugänge zur digitalen Fortbildung. Eine qualitative Studie mit Dermatologen, Pflegefachpersonen und Patienten in der Dermatologie identifizierte eine geringe digitale Gesundheitskompetenz der Patienten als zentrale Barriere für die digitale Transformation in der dermatologischen Versorgung.^18^ Dies unterstreicht auch den Weiterbildungsbedarf auf Seiten der Patient*innen.

Diese Umfrage war die erste, die sich systematisch mit der Nutzung von digitalen Gesundheitsanwendungen (DiGA) und Large Language Models (LLMs) in der Dermatologie befasste. Die vorliegende Studie weist mehrere Limitationen auf, die bei der Interpretation der Ergebnisse berücksichtigt werden sollten. Die Hauptlimitation der Studie besteht in der vergleichsweise kleinen Stichprobengröße von 100 Teilnehmenden, die nicht repräsentativ für die Gesamtheit der dermatologischen Ärzteschaft ist. Gleichzeitig war die befragte Gruppe von Dermatologen vergleichsweise jung. Dass gerade die jüngeren Teilnehmenden ihr Wissen als eher gering einschätzen und einen hohen Fortbildungsbedarf sehen, unterstreicht die besondere Relevanz. Frühere Studien zeigen, dass insbesondere jüngere Fachkräfte digitale Tools deutlich häufiger in der klinischen Routine einsetzen.^19^ Ebenfalls erfolgte die Erhebung im Rahmen freiwilliger Fortbildungsveranstaltungen. Daraus ergibt sich eine potenzielle Verzerrung, da junge, digital affine oder fortbildungsbereite Dermatologen möglicherweise überrepräsentiert sind.

## SCHLUSSFOLGERUNG

Trotz einer überwiegend positiven Bewertung gegenüber digitalen Technologien, insbesondere DiGA, finden diese bislang nur begrenzt Anwendung in der dermatologischen Praxis. Die Umfrageergebnisse zeigten neben einem klaren Fortbildungsbedarf auch strukturelle und organisatorische Barrieren, die einer breiten Implementierung im Versorgungsalltag entgegenstanden. Um das vorhandene Potenzial digitaler Anwendungen zur Verbesserung der dermatologischen Versorgung zu realisieren, sind gezielte Qualifizierungsangebote, dermatologische DiGA, andere angemessene Vergütungsmodelle und praxistaugliche Unterstützung bei der Implementierung erforderlich.

## FINANZIERUNG

Die Studie wurde durch Hausmittel des Instituts für Digitale Medizin finanziert.

## DANKSAGUNG

Wir möchten uns bei allen Ärzt*innen herzlich für die Teilnahme bedanken.

Open access funding enabled and organized by Projekt DEAL.

## INTERESSENKONFLIKT

Keiner.
